# Lessons learned, challenges and outlooks for decision-making after a decade of experience monitoring the impact of indoor residual spraying in Benin, West Africa

**DOI:** 10.1186/s12936-020-3131-1

**Published:** 2020-01-28

**Authors:** Martin C. Akogbéto, Fortuné Dagnon, Rock Aïkpon, Razaki Ossé, Albert S. Salako, Idelphonse Ahogni, Bruno Akinro, André Sominahouin, Aboubakar Sidick, Filémon Tokponnon, Germain G. Padonou

**Affiliations:** 1 Centre de Recherche entomologique de Cotonou (CREC), Cotonou, Benin; 2 Faculté des Sciences et Techniques, de l’ Université d’Abomey-Calavi, Cotonou, Benin; 3 US President’s Malaria Initiative, US Agency for International Development, Cotonou, Benin; 4 Technologies, Ingénierie et Mathématiques, Université Nationale des Sciences, Abomey, Bénin; 5 Université Nationale d’Agriculture, de Porto-Novo, Bénin; 6 Faculté des Sciences Humaines et Sociales, de l’ Université d’Abomey-Calavi, Cotonou, Benin; 7 National Malaria Control Program (NMCP), Cotonou, Bénin

**Keywords:** IRS, Lessons learned, Challenges, Outlooks, Benin

## Abstract

**Background:**

Since 2008, Indoor Residual Spraying (IRS) has been performed in Benin in 19 districts, including 4 in southern Benin, 9 in Atacora, and 8 in Atacora, Alibori and Donga in northern Benin. However, Benin still struggles with questions about IRS cost–benefit and epidemiological impact. Lessons learned and challenges from 10 years of IRS in Benin to be shared with the stakeholders involved in vector control implementation for decision-making.

**Methods:**

Entomological parameters have been assessed entomological parameters in IRS communes since 2008. In all IRS intervention communes, decreases in human biting rate (HBR) of *Anopheles gambiae*, blood feeding inhibition and entomological inoculation rate (EIR) as compared to control district have been measured.

**Results:**

EIR was reduced by 80–90%, which is encouraging, but should be observed with caution because: (i) the reduction may be insufficient to decrease epidemiological indicators given that the residual EIR in IRS districts is still higher than it is in some regions of stable malaria; (ii) the reduction in EIR is based on comparisons with control communes, but it is difficult to select control areas with the same environmental characteristics as intervention areas; (iii) despite the reduction, half of all mosquitoes that entered IRS-treated houses succeeded in taking human blood meals. Further, there are behaviours among Benin’s population that limit IRS efficacy, including recent data showing that > 90% of people are not protected by IRS between 7 and 10 p.m. This is due to the fact that they remain outdoors and that most people are not protected from mosquito bites after 10 p.m. because they either sleep outdoors without IRS protection or indoors without an ITN. Moreover, people have large amounts of clothing hanging on walls where mosquitoes can rest instead of IRS-treated walls. Finally, other components are important to consider in implementing IRS among which: (i) Vector resistance management strategies are sometimes poorly understood; this is actually different from the need to replace one insecticide with another after the emergence of resistance; (ii) African countries should prepare to finance IRS themselves.

**Conclusion:**

To curtail residual malaria transmission, additional interventions able to target vectors escaping IRS should be prioritized.

## Background

**Fig. 1:**
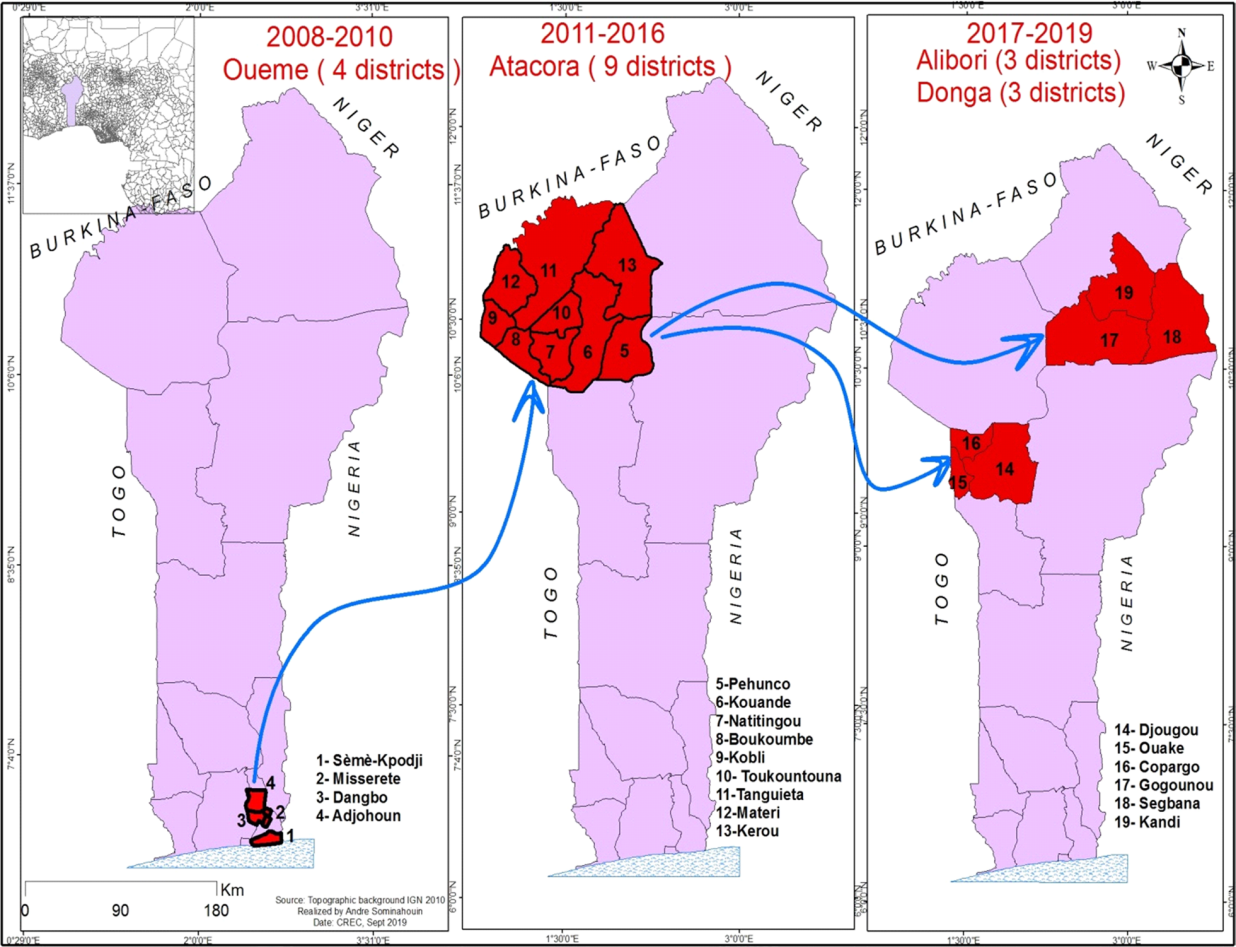
In 10 years, IRS Benin was withdrawn and woved from Ouémé to Atacora, and from Atacora to Alibori/Donga

**Fig. 2:**
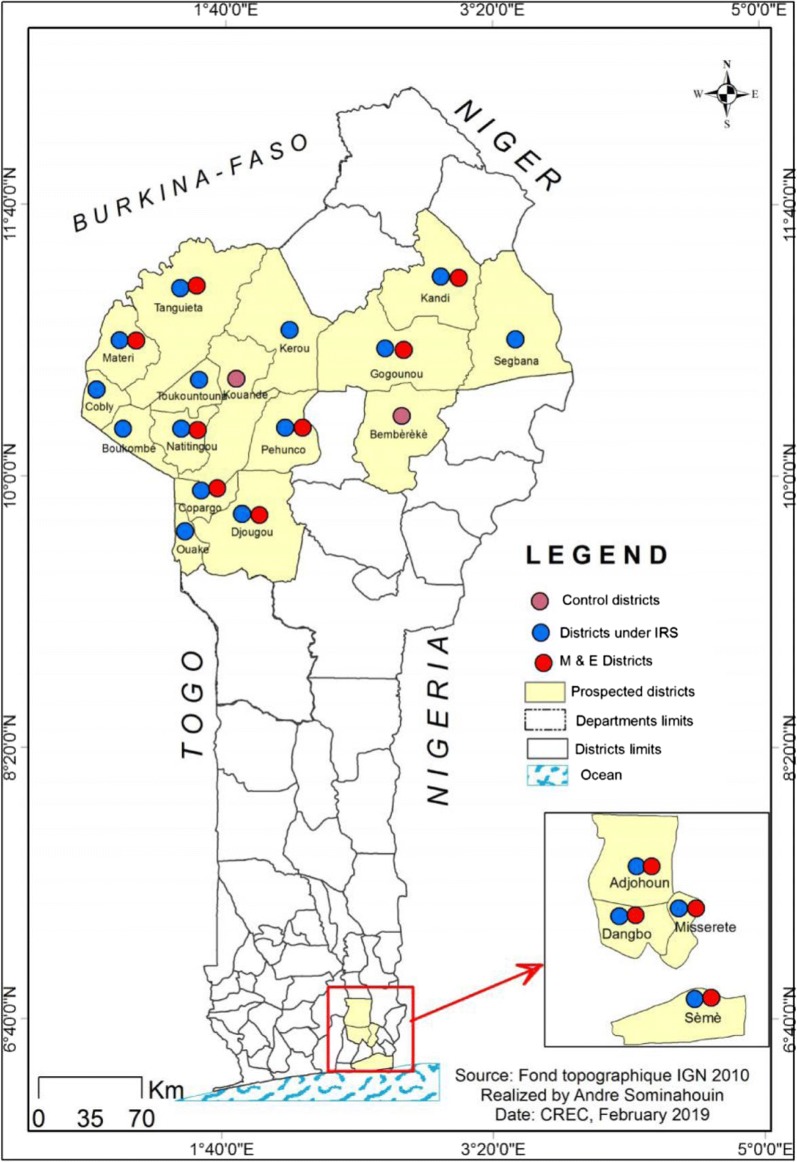
Map of Benin showing the districts under IRS and those under M&E and control in Benin

For some years, impregnated materials have been adopted and used as the main prevention tool against *Anopheles* mosquito bites in Africa, so as to reduce the burden of malaria morbidity and mortality. In 2008, indoor residual spraying (IRS) was introduced in Benin on a large scale in order to complete the action of long-lasting insecticidal nets (LLIN). The first campaign of IRS was implemented in four communes in Ouémé region **(**Adjohoun, Akpro-Missérété, Danbgo, Sèmè-Kpodji) in southern Benin for 3 years (2008–2010). In 2011, this intervention was withdrawn for the Atacora region in the northern Benin (Fig. 1). For 6 years (2011–2016), people of the 9 districts of this region were protected against mosquito bites. From 2017 to 2018, Alibori and Donga regions (northern Benin), and 2 Atacora districts (Kerou and Pehunco) benefited from IRS protection (Fig. 1). Today, a total of 19 districts of Benin have been involved in the implementation of IRS (Fig. 2) [1–4].

As documented in recent World Malaria Reports, reductions in malaria disease burden have coincided with the massive scale-up of malaria prevention measures, particularly the use of LLINs and IRS [5, 6]. Some African countries (Zimbabwe, Namibia, Swaziland, Bostwana and Mozambique) have had a reduction in malaria transmission and parasite rates in children [7] because of the IRS strategy.

IRS implementation has been increased in Africa to strengthen the action of the existing LLINs as first prevention strategy against malaria. The proportion of African population at risk of malaria but protected by IRS increased from 5% in 2005 to 11% in 2011 [5, 6, 8]. In a recent past, more than 20 countries (Benin, Burkina Faso, Burundi, Cambodia, Democratic Republic of Congo, Ethiopia, Ghana, Kenya, Liberia, Madagascar, Malawi, Mali, Mozambique, Nigeria, Rwanda, Senegal, Sierra Leone, Tanzania, Uganda, Zambia and Zimbabwe) were involved in IRS implementation. Today, 3 other countries (Cameroon, Côte d’Ivoire and Niger) joined the group with the support of President’s Malaria Initiative (PMI) of US Government. Most of African countries have now signed up to the Millennium Development Goals (MDGs), whose components include a 75% reduction of malaria morbidity and mortality between 2015 and 2030 [9]. To achieve this level of malaria reduction within 15 years, an integrated control based on the simultaneous use of multiple strategies against the vector and the parasite is necessary.

In Benin, IRS and LLINs have resulted in drastic reductions in entomological parameters, particularly entomological inoculation rate (EIR) [10, 12, 13] that have not necessarily been associated with similar reductions in epidemiological indicators. In Atacora, routine health facility data did not suggest a significant reduction in the number of malaria cases despite being an area that has received annual IRS for six years. This situation explains why Benin still struggles with questions about IRS cost–benefit and epidemiological impact. The question is not strange because some articles on the efficacy of LLINs and IRS in 5 countries (Benin, Cameroon, Kenya, India and Sudan) [14–17] and in Tori, a village in the southern Benin [18, 19] where *Anopheles* mosquitoes are resistant to pyrethroids showed mitigated results based on little correlation between the decrease of entomological parameters and parasitological and clinical parameters.

After a decade of experience monitoring the impact of IRS in Benin, some lessons learned can be shared with stakeholders involved in vector control, particularly the National Malaria Control Programmes and partners (PMI/USAID and others).

A paper published by Akogbeto et al. [13] summing up six years of experience of implementation of IRS in Benin, had discussed new challenges and future prospects for its success. Four years later, IRS was implemented and followed in other environments. In addition, since 2017, monitoring and evaluation (M&E) of IRS has identified practices of households, which might not be supportive of good and effective vector control. For example, populations may not benefit from IRS protection inside their homes because they sleep outdoors.

## Methods

### Study area: regions and districts covered by IRS

The first campaign of IRS was implemented in the region of Ouémé, situated in the south of Benin. This region is characterized by two dry seasons (December to March and August to September) and two rainy seasons (April to July and October to November). The existence of two rainy seasons in the year results in an almost year-round presence of *Anopheles* and permanent malaria transmission. In contrast to that, the regions of Atacora, Alibori and Donga are characterized by one dry season (November to May) and one rainy season (June to October). Four districts were covered by IRS in Oueme (Adjohoun, Dangbo, Missérété and Sèmè) and a district of Porto-Novo served as control (Figs. 1 and 2). In Atacora, nine districts (Natitingou, Tanguieta, Toucountouna, Materi, Boucombe, Cobly, Kouandé, Péhunco, and Kerou) were covered by the IRS, but only four were used for monitoring (Natitingou, Tanguieta, Kouandé and Pehunco) and one for control (Copargo). In Alibori and Donga regions, three districts were concerned by IRS: Kandi, Gogonou, Segbana (in Alibori), and Djougou, Copargo, Ouaké (in Donga). The M&E was implemented in Kandi and Gogounon, and in Djougou and Copargo, with Bembèrèkè and Kouandé as controls, respectively for Alibori districts and Donga (Figs. 1 and 2). Copargo served as control from 2011 to 2016 when Atacora region was under IRS, but as treated commune from 2017 during the Alibori and Donga IRS implementation.

**Fig. 3:**
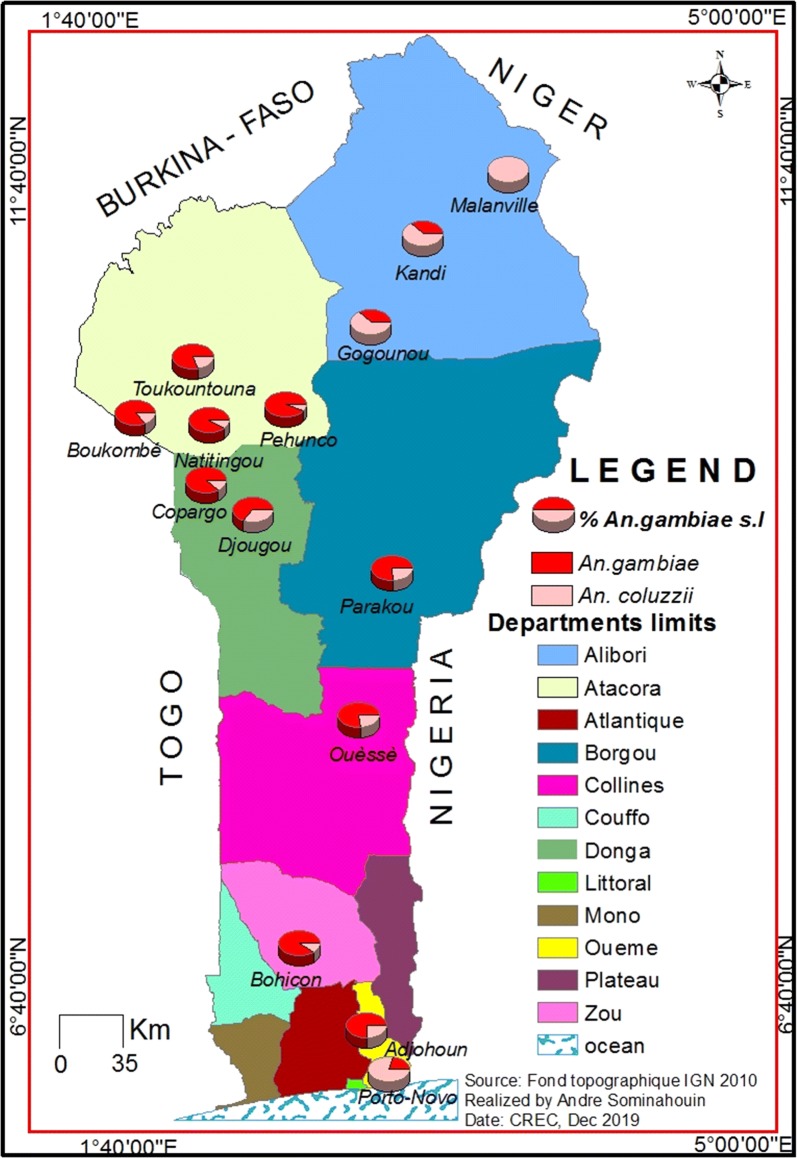
Map of Benin showing the two main vectors involved in malaria transmission in Benin: *Anopheles gambiae* and *Anopheles coluzzii*

In Atacora, Alibori and Donga, the major economic activity is agriculture, mainly cotton and millet, for which various classes of pesticides (pyrethroid, carbamate, organophosphate) are used. In contrast, the main activities in Ouémé include trade, corn agriculture and fishing facilitated by the presence of Ouémé River. The M form of *An. gambiae,* recently named *Anopheles coluzzii* [20], is the major malaria vector in Ouémé and the S form named *An. gambiae* [20, 21] in Atacora, Alibori and Donga (Fig. 3). During the IRS implementation, the coverage rate was more than 80% everywhere [2, 4].

**Fig. 4:**
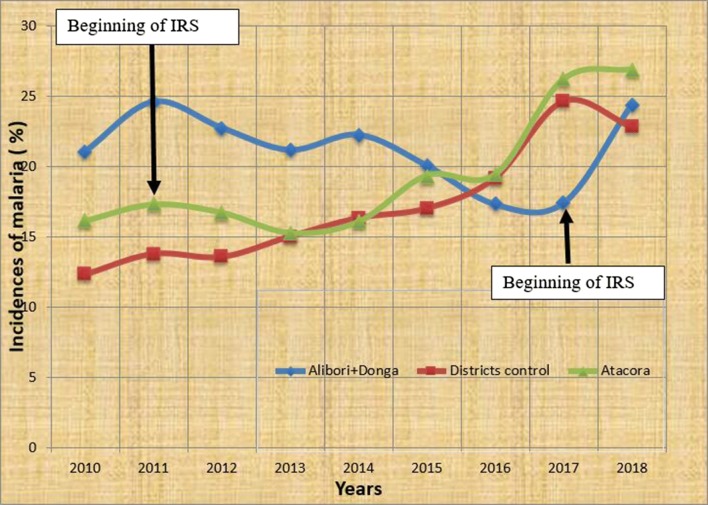
Annual incidences of malaria in Alibori-Donga’s departments and control communes (Kouandé, Bembèrèkè)

Entomological data were collected in each M&E district in two villages, one in the central part, and one in the peripheral part. LLINs were distributed every 3 years throughout the mass campaign since 2011 in the four regions. Malaria health data come from the database of the Statistical office of health zones and cover the period from 2010 to 2018. Only the incidence attributed to malaria was used in this study (Figs. 4 and 6).

### Intervention partners and insecticides used

All the IRS campaigns were supported by the President’s Malaria Initiative (PMI), funded by the US Government. The technical implementation was conducted by two technical partners: Research Triangle International from 2008 to 2011 and Abt Associates (VectorLink) from 2012 to 2018. The M&E on the effectiveness of the intervention was carried out by “Centre de Recherche Entomologique de Cotonou”, one of the research institutions of the Ministry of Health in Benin.

Due to the multiple resistance of *An. gambiae* to all classes of insecticides in Benin [10–14], IRS campaigns were preceded by a study in experimental huts in order to select the best insecticide to be used. Various insecticides were evaluated: Sumithion^®^ 40 WP (fénitrothion, an organophosphate), Master Quick ZC (mixture of chlorpyrifos 250 g/l + deltamethrin 12 g/l), and Ficam M^®^ (a carbamate: bendiocarb 800 g/kg). Bendiocarb (800 g/kg) containing product was selected from 2008 to 2012 for house spraying in Ouémé and Atacora. After two years of bendiocarb use in Atacora, the emergence of *An. gambiae* resistance to this product was reported [4, 11, 13, 18, 19]. Then, a second evaluation was conducted and Ficam M^R^ was rotated with Actellic 50 EC, an organophosphate (pirimiphos methyl 50 EC) in 2013 and Actellic 300 CS from 2014 to 2018 in the regions of Atacora, Alibori and Donga.

During IRS implementation, bendiocarb or pirimiphos methyl is applied once a year, at the beginning of the rainy season which corresponds to the period of high malaria transmission (June-October). However, in 2010, two treatments were carried out in southern Benin, one at the beginning of the long rainy season (April-July), and the other at the beginning of the short rainy season (September–October).

### Adult mosquito collections methods

The same protocol was globally followed from 2008 to 2018 in the 4 regions. To determine mosquitoes resting on walls inside the houses and those exiting, exit traps (EWT) have been installed at the windows of some sleeping rooms in Ouémé region. Mosquitoes that entered the houses at night were retained in the traps while exiting at dawn and then caught in the morning. Mosquitoes which did not enter the exit window traps were caught after space spraying by Pyrethrum Spray Catch (PSC) inside the houses. In each district, 2 villages were selected for collection of residual fauna of mosquitoes: one in the central part of the district, and one in the peripheral part. In each village, 10 to 20 bedrooms were selected. The number depended on the availability of the eligible bedrooms, the willingness of the owners to collaborate with the team and to participate in the study. The number of unfed, fed, half gravid and gravid mosquitoes collected by PSC and EWT in treated and control rooms were registered and compared. To determine the impact of IRS on indoor resting behaviour of mosquitoes the proportion of unfed, fed, gravid and half gravid females in districts under IRS and those in control districts was compared. On the one hand, the behaviour of mosquitoes found indoors and collected by PSC is regarded as an endophilic behaviour; but in this study, this characteristic is determined by considering only gravid and half gravid mosquitoes. On the other hand, mosquitoes which are collected in the EWT are considered to show an exophilic behaviour.

In each intervention district under M&E and control districts, adult mosquito collections were carried out through human landing catch (HLC). Two villages were selected, and two houses were chosen per village for mosquito collection to monitor malaria transmission. Monthly (every month during the first 6-month period following the spray) and bi-monthly (every 2 months during the second 6-month period), mosquito collections were carried out by HLC from 9 pm to 6 am indoors and outdoors using a mouth aspirator, by resident volunteers who had previously given consent. Every 2 months, two nights of mosquito collections were carried out.

Before implementing M&E of IRS in Ouémé, Alibori, Atacora and Donga, the hourly biting rate of mosquitoes was monitored in various communes. This investigation has showed that, contrary to *Culex quinquefasciatus* and *Mansonia africana*, *An. gambiae* feeding rate was too low before 9 pm. This is why the collection team preferred to start HLC from 9 pm.

### Mosquito identification and processing

All female mosquitoes belonging to *An. gambiae* complex were identified based on morphological characteristics using standard identification keys [22]. About 40–50% of the *Anopheles* vectors captured through HLC were dissected to assess their physiological age [23].

### Molecular analyses

Members of *An. gambiae* s.l. were identified to species by polymerase chain reaction (PCR), following the protocols developed by Santolamazza et al. [24]. Head-thoraxes were tested using enzyme-linked immunosorbent analysis (ELISA) according to Wirtz et al. [25] for the presence of circumsporozoite protein (CSP) of *Plasmodium falciparum*, the major malaria parasite occurring in the study area. The recorded HLC data were used to assess the feeding behaviour (HBR) of mosquitoes and the EIR of the vectors.

### Estimation of entomological parameters

Various entomological parameters were analysed:

1. Reduction of mosquito entry in districts under IRS intervention compared to control districts;
2. Resting behaviour of mosquitoes in treated houses related to control houses;
3. Induced exophily (the proportion of mosquitoes which exit the treated houses early and are found in exit window traps) compared to the natural exophily; The percentage of exophilic mosquitoes is the proportion of mosquitoes which entered the houses at night, and caught in the morning after being retained in the traps while exiting at dawn (proportion [PSC + EWT] − PSC).
4. Blood-feeding inhibition due to the presence of insecticides on the walls (blood feeding of *An. gambiae* in the houses under IRS compared to that in the control houses). The blood feeding rate is the ratio between the number of mosquitoes which have taken blood on their hosts and the total number of mosquitoes collected. The endophilic mosquitoes are considered as gravid +half gravid mosquitoes resting in the houses and collected by PSC and EWT.
5. The sporozoïte rate is the number of *P*. *falciparum (*CSP) positive mosquitoes divided by the total number of analysed mosquitoes.
6. The HBR is expressed as the number of bites per person per night (b/p/n). This rate is obtained by dividing the number of mosquitoes collected by the number of volunteers, with reference to the number of rounds.
7. The EIR is estimated by multiplying the sporozoïte rate by the human-biting rate.

### Data analysis

The data were analysed with the statistical R software, version 2.8. The Poisson method was used to estimate and compare the confidence intervals of vector density (exophily, endophily) and EIRs of *An. gambiae* s.l. [26]. The Chi^2^ test of comparison of proportions was used to compare blood feeding rate, sporozoite index of *An. gambiae* s.l. These different parameters were compared between the treated and control areas.

### Ethical considerations

The protocol of this study has been reviewed and approved by the Institutional Ethics Committee of Cotonou Entomology Research Center (Grant No IORG005698). Verbal consent was obtained from local mosquito collectors before being involved in the study. They subsequently received a vaccine against yellow fever as a prophylactic measure. An agreement with health facilities close to sites was also obtained for the free anti-malaria treatment of mosquito collectors who might suffer from malaria.

## Results

### Vectors involved in malaria transmission in the four regions

*Anopheles gambiae* s.l. is the abundant *Anopheles* collected in the four regions. *Anopheles funestus* is present but at very low frequency. *Anopheles coluzzii* and *An. gambiae* are the two important species of *An. gambiae* complex. The two species were collected in the majority of the districts under IRS and control (Fig. 3).

### Residual density, exophily/endophily and blood feeding rate of *An. gambiae* s.l. in districts under IRS and control districts

#### In Alibori and Donga regions

In 2017 and 2018, the number of *An. gambiae* s.l. collected indoor in the treated houses was 3 times lower compared to untreated houses (control). As a matter of fact, during the period of June-December 2017 (first round of IRS), the room density (number of *An. gambiae*/bedroom) was 0.33 (299/911) in IRS areas and 1.19 (320/270: 3.6 times higher) in the control areas (p < 0.001). During the second round, the results were the same: 0.38 *An. gambiae* per bedroom (121/322) against 1.40 (165/118: 3.7 times higher) in the control p < 0.001) (Table 1). On the other hand, only 5 gravid and half gravid *An. gambiae* were collected from a total of 299 during the first round of IRS (2017), that is to say a very low rate of endophily (1.6%) in the IRS districts against 19.4% (62/320) in untreated districts (p < 0.05). However, for the second round (2018), during the same period (June-December), the endophily rate was low in both treated districts (8.2%:10/121) and in untreated districts (7.8: 13/166). Regarding the blood feeding behaviour, a lower rate (63.87%: 191 fed *An. gambiae*/299) was registered in 2017 in IRS districts compared to the control districts (74.37%: 238/320) (Table 1) (p = 0.0005). During the second round of IRS (2018), the two rates were similar: 74.4% (90/121) and 77.7% (129/166) (p = 0.452) (Table 1). When the abdomens of the fed mosquitoes were analysed by ELISA, the majority was fed on human: 76.81% (53/69) on human and 16% (11/69) on human + cattle in the IRS areas and 87.71% (51/57) on human, 1.7% (1/57) on human + pig, 8.7% (5/57) on cow and, 1.7% (1/57) on human + goat in control area.

**Table 1:** Residual density and blood feeding rates of *An. gambiae* s.l. collected in districts under IRS and control districts after 2017 and 2018 IRS campaigns in Alibori and Donga

Period	Districts	Nb of room	Nb *An. gambiae* (s.l.) collected	Density/Room	Unfed	Fed	Gravid	Half-Gravid	Blood feeding rate %	P- value
June 2017–Dec 2017	Kandi	246	47	0.19	16	31	0	0	65.96	0.043
	Gogounou	239	114	0.48	45	67	1	1	59.65	0.0001
	Bembèrèkè (control)	210	184	0.88	13	139	22	10	80.98	–
	Djougou	223	54	0.24	21	32	0	1	61.11	0.105
	Copargo	203	84	0.41	21	61	0	2	75	1
	Kouande (control)	60	136	2.27	7	99	28	2	74.26	–
	Total treated districts	911	299	0.33	103	191	1	4	65.22	0.0005
	Total control districts	270	320	1.19	20	238	50	12	78.13	–
June 2018–Nov 2018	Kandi	80	28	0.35	2	22	3	1	82.14	1
	Gogounou	80	21	0.26	4	17	0	0	80.95	1
	Bembèrèkè (control)	78	126	1.62	21	96	4	5	80.16	–
	Djougou	82	22	0.27	3	19	0	0	86.36	1
	Copargo	80	50	0.63	12	32	4	2	68	0.119
	Kouande (control)	40	39	0.98	3	33	4	0	84.62	–
	Total treated districts	322	121	0.38	21	90	7	3	76.86	0.452
	Total control districts	118	165	1.4	24	129	8	5	81.21	–

May, IRS implementation month; June-November/December, period of residual effect of pirimiphos methyl

P-value: P-value of comparison of the blood feeding rate of *An. gambiae* between the treated and control districts

#### In Atacora region

During the period when pirimiphos methyl was available and active on the treated walls (June to November 2014), 36% (63/172) of *An. gambiae s.l*. were collected by PSC indoors and 64% (109/172) in EWT. In the control areas, the percentage of *An. gambiae* collected indoors by PSC was 2 times higher: 70% (204/290) by PSC against 30% (86/290) in EWT. The exophily rate was significantly higher in IRS areas than in control areas (p < 0.05). An inhibition of the blood feeding rate was also observed in the treated areas: 45.3% (78/172) against 76.5% (222/290) in the control (p < 0.95).

#### In Ouémé region

In Ouémé, two periods were followed: database period (before IRS campaign) and intervention period. From a total of 928 *An. gambiae s.l*. collected indoors by PSC and EWT before IRS (control), 526 fed mosquitoes and 348 gravid and half gravid mosquitoes have been registered, i.e., a blood feeding rate of case 56.7% (526/928) and an endophily rate of 37.5% (348/928). A total of 305 *An. gambiae* s.l. was collected in EWT, which means an exophily rate of 32.86%. During IRS intervention period, very few *An. gambiae* s.l. was found: 89 indoors for the same number of houses: a reduction of 90% ([928-89]/928) of entry of *An. gambiae s.l*. in the treated houses. The blood feeding rate decreased from 56.7% to 25.8% (23 fed *An. gambiae*/89), did the endophily rate (4.5%: 4/89). Also, an increase in exophily was noticed: 95.5% (85/89) against 32.86% for the natural exophily, i.e. an induced exophily of 62.64% due to insecticide on the walls.

### Sporozoite index, entomological inoculation rate and parous rate in districts under IRS and control districts

**Fig. 5:**
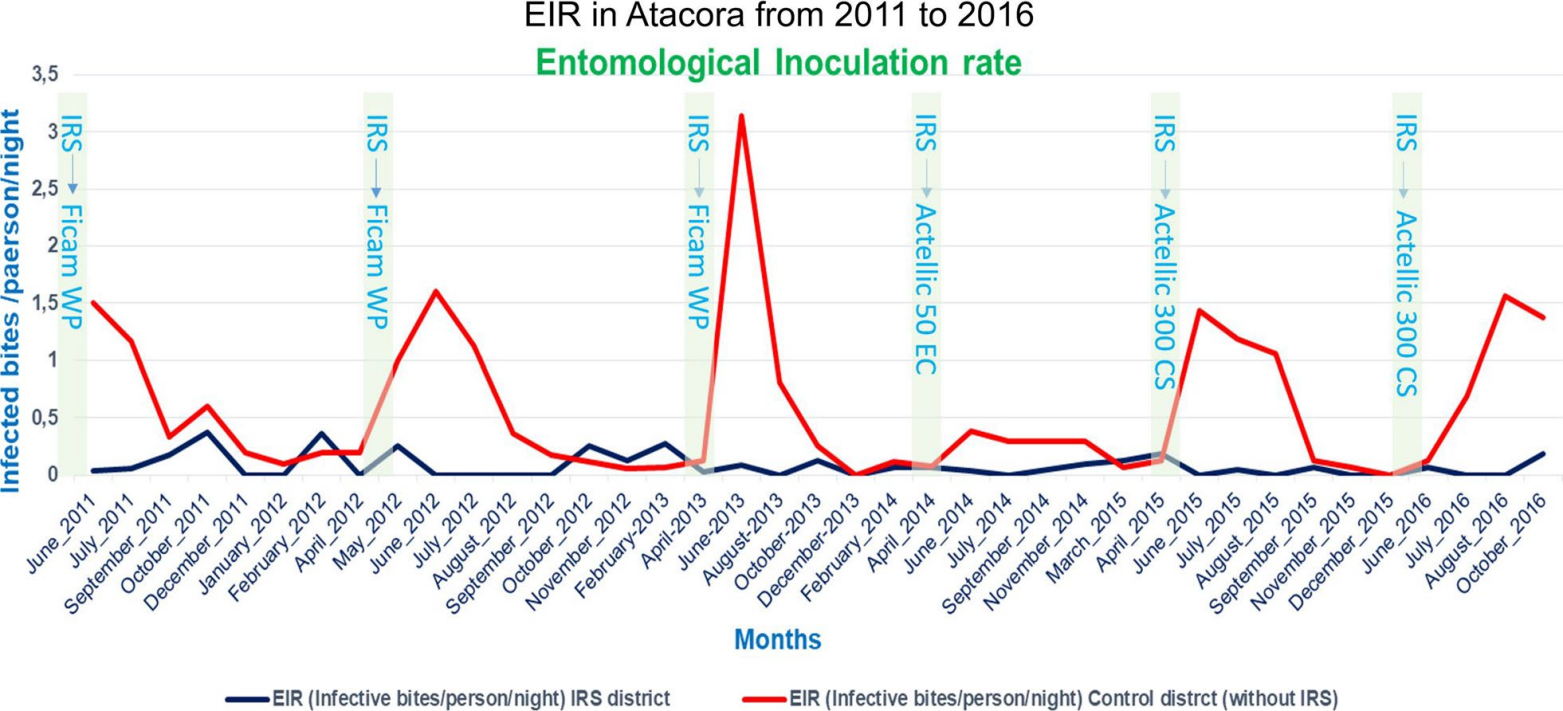
Dynamic of EIR after IRS implementation in the region under IRS in Atacora region and in control communes

**Fig. 6:**
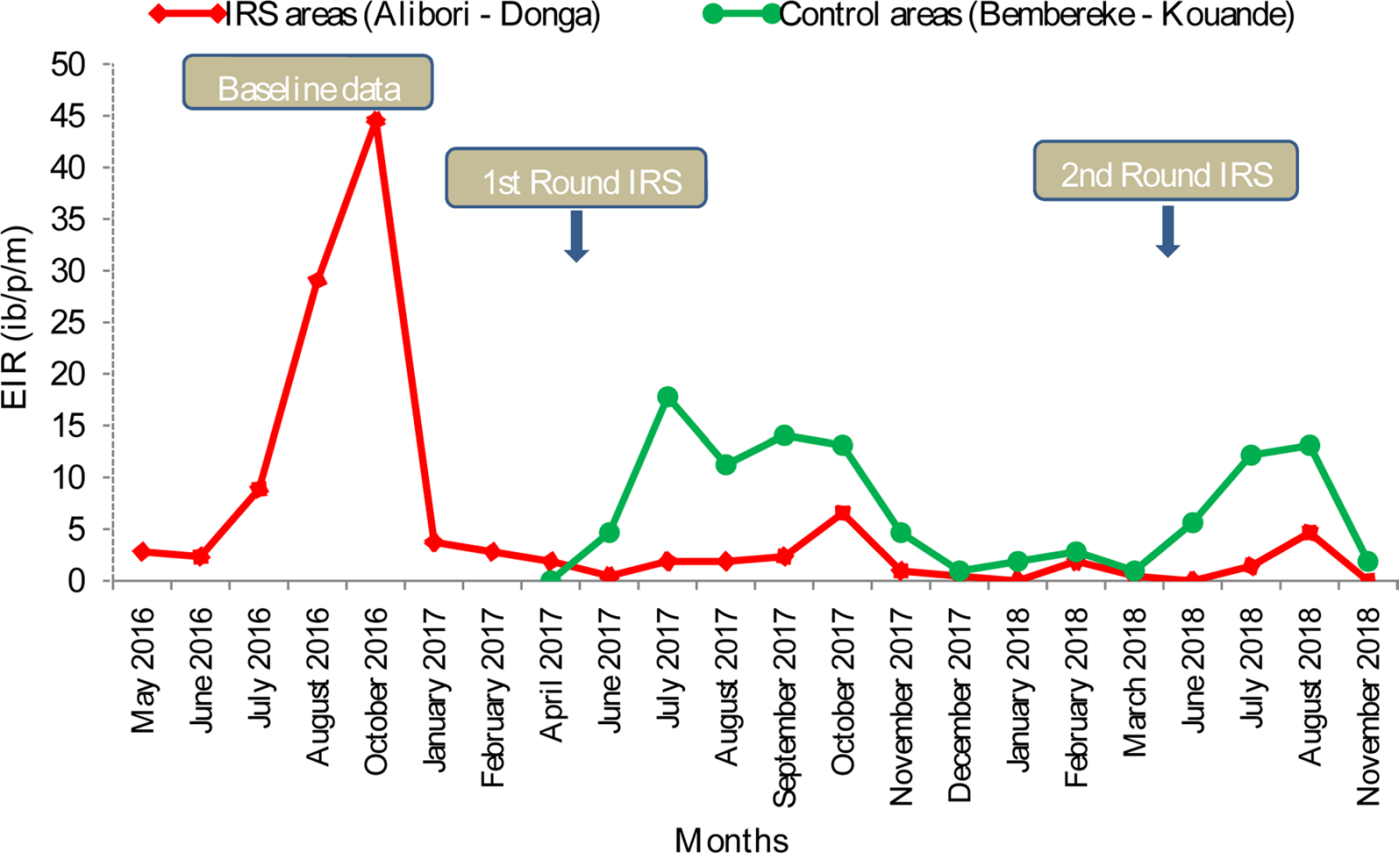
Dynamic of EIR after IRS implementation in the regions under IRS (Alibori, Donga) and in control communes (Bembereke, Kouande)

The EIR indicates the intensity of malaria transmission at a given moment. It is based on 2 important parameters: the human biting rate (HBR = ma) and the sporozoite index (*Plasmodium* circumsporozoite antigen). Data registered for 10 years were numerous. Some data collected in Ouémé and Atacora were published in 3 PhD theses (Padonou [2] and Ossè [3] in Ouémé, and Aïkpon [4] in Atacora) and many papers [10–13]. In the current paper, the intention is not to repeat the already published results of EIR obtained in the regions of Ouémé and Atacora, globally characterized by a drastic decrease and a low parous rate of *An. gambiae* in all districts under intervention. Figure 5 shows the dynamics of EIR from June 2011 to October 2016 in Atacora region. The lowest EIRs were observed during the dry periods (December to April in both treated and control areas. After IRS implementation, lower monthly EIRs were observed in the treated areas compared to the control areas between June and September/October which equals to 3 or 4 months of impact each year. From 2016, in the new regions (Alibori and Donga), IRS implementation was preceded by one year of source data collection. Results obtained were indicated in Fig. 6 and in Table 2. Due to EIR variation in the course of the year, results were presented in several phases based on the period of PM residual effect. Four phases were considered:

**Table 2:** Human biting rate (HBR), sporozoite index (SI %), entomological inoculation rate (EIR) and parous rate in *Anopheles gambiae* in communes under IRS and control commune according to the period of residual effect of Pirimiphos methyl (PM) on treated walls

Periods	Indicators	Districts under IRS (Alibori, Donga)	Control district (Bembereke, Kouande)	P-value
Period of residual effet of Actellic CS (June–Sep 2017)	HBR/night	7.56	10.81	< 0.0001
	SI (%)	0.999 (19/1902)	4.18 (55/1316)	< 0.0001
	EIR/month	2.266	13.551	< 0.0001
	Parity rate (%)	36.62 (364/994)	57.24 (257/449)	< 0.0001
After residual effet of Actellic CS (Oct–Dec 2017)	HBR/night	2.77	3.2	0.043
	SI (%)	2.92 (18/616)	6.42 (21/327)	0.016
	EIR/month	2.424	6.161	< 0.0001
	Parity rate (%)	49.33 (148/300)	58.16 (114/196)	0.066
Before IRS 2018 (Jan–Mar 2018)	HBR/night	1	1.45	0.002
	SI (%)	2.72 (9/331)	3.95 (9/228)	0.572
	EIR/month	0.816	1.717	< 0.05
	Parity rate (%)	76.97 (117/152)	71.70 (76/106)	0.415
Period of residual effet of Actellic CS (June–Aug 2018)	HBR/night	7.76	11.01	< 0.0001
	SI (%)	0.81 (11/1358)	2.6087 (27/1035)	0.0008
	EIR/month	1.885	8.618	< 0.0001
	Parity rate (%)	48.36 (353/730)	62.96 (289/459)	0.000117

Period of residual effect of pirimiphos methyl: when the mortality of *An. gambiae* Kisumu is ≥ 80% after exposure to tread walls

- during (June–September) and after (October-December) the period of pirimiphos methyl residual activity on the treated walls (2017, first round);
- before (January–March) and during (July–August) the period of pirimiphos methyl residual activity as part of the second IRS campaign (2018).

Each year, IRS was performed in May before the beginning of the rainy season. The period of residual activity is determined after performing WHO cone bioassay on the treated walls using a susceptible strain of *An. gambiae* (Kisumu strain).

Table 2 shows the variation of HBR, sporozoite index (SI) of *An. gambiae* s.l. for circumsporozoite protein, EIR and parous rate of *An. gambiae* during the four phases. During the first round (2017) in Alibori and Donga, when pirimiphos methyl was active on the walls in the intervention districts, the positivity of *An. gambiae* for *P. falciparum* circumsporozoite antigen (CS+) was low (0.99%: 19 thoraces+/1902) compared to control district (4.18%: 55th+/1316) (p < 0.0001) (Table 2). During the second round in 2018, similar variation was observed, respectively: 0.81% (11th+/1358) against 2.60% (27/1035) (p < 0.0001). Despite the loss of the residual activity of pirimiphos methyl, 6 months after the treatment of the walls, the positivity of *An. gambiae* for *P. falciparum* circumsporozoite antigen was low in the districts covered by IRS: 2.92% (18 th +/616) against 6.42% (21th+/327) in control district in 2017 (p = 0.016) (Table 2). Similar results were observed 9 months after the spraying: 2.72% (9th+/331) in districts under IRS against 3.95% (9th+/228) in control district (p = 0.572) (Table 2). In parallel, during the first period of the residual activity of pirimiphos methyl (June -September 2017), EIR was 3 times lower in the districts under intervention (2.26 infected bites of *An. gambiae* per human per month) compared to the control districts (13.55 infected bites of *An. gambiae* per human per month), which means a reduction of 83.3% in 2017 (p < 0.0001) (Table 2 and Fig. 6). In the same period of residual activity of pirimiphos methyl in 2018, the reduction of EIR in treated districts was important as well: 78.16% (8.61 infected bites/human/month against 1.88) (Table 2 and Fig. 6). Six and 9 months after the spraying, high reductions were also observed in districts under IRS, respectively, 60.7% (6.14 vs 2.42) and 52.6% (1.71 vs 0.16) (Table 2 and Fig. 6).

The high reduction of EIR in the intervention areas was in line with the decrease of the longevity of *An. gambiae* in these areas: parous rate = 36.62% (364/994) in June–September 2017 against 57.24% (257/449) in the control district (Table 2).

## Discussion

### The strengths of the IRS campaigns in Benin

The four strengths of the IRS campaigns in Benin which caught the authors attention of this paper and have already been mentioned [13] were: (i) the great support from the population; (ii) the skill and expertise of the international NGOs responsible for implementation; (iii) the high coverage of protected households exceeding 80% of the sleeping quarters; and, (iv) a significant reduction in malaria transmission. The drastic decrease of the EIR mentioned from 2008 to 2016 in Ouémé and Atacora was also observed from 2017 to 2018 in Alibori and Donga. This article does not aim at emphasizing these results. It rather presents the shortcomings related to the IRS monitoring which remained silent about concerns raised by Benin NMCP and the partner funding IRS in Benin, the challenges and attempts to suggest solutions. Beside the suggestions already mentioned [13] in relation with appropriate locations for IRS implementation and management of *Anopheles* resistance to insecticides, authors would like to draw attention to challenges affecting IRS impact.

### Challenges affecting IRS impact

#### Challenge related to the residual malaria transmission in communes under IRS: lack of evident correlation between the decrease of EIR and malaria incidence

Despite the coverage of more than 80% of the structures by IRS and a drastic reduction of EIR, there is still significant residual malaria transmission in the four regions. In Ouémé region, despite two rounds of IRS in 2010 and the reduction of 90% of EIR, the 10% remaining representing the residual malaria transmission should be considered relatively high. Therefore, despite IRS in Adjohoun, Dangbo and Sèmè in Ouémé region, this residual transmission was estimated between 12.91 and 55.14 *An. gambiae* infected bites per year [2]. Theoretically, in this region, despite IRS, everybody under protection of IRS was at risk of being in contact with 1 to 4 *An. gambiae* carrying *P. falciparum* in the salivary glands per month. The same situation was noticed in Alibori and Donga where a residual transmission of around 2 infecting bites per month after 80% reduction of EIR occurred; this was also the same in Atacora only in 2011 [4]. In view of the complaints from the stakeholders about the lack of evidence of IRS epidemiological impact and the absence of significant reduction in the number of malaria cases suggested by the routine health facility data although these areas have been under annual IRS for many years, the reduction of 80–90% of EIR is encouraging, but should be observed with caution: it may be insufficient to decrease epidemiological indicators given that the residual EIR in IRS communes was still higher than it was in some regions of stable malaria. However, EIR calculated for the communes under IRS might be overestimated. As a matter of fact, bendiocarb and pirimiphos methyl make mosquitoes fall from treated walls among which most of them blood fed. Since the houses of the study area were not surrounded by ant traps, such mosquitoes could be removed before PSC mosquito collections carried out by the technicians of CREC. Thus, the number of mosquitoes entering the houses and dead after blood meal might be underestimated. However, as EIR was calculated using mosquitoes collected using HLC, the number of mosquitoes which could escape to the ability of mosquito collectors would be low. Regarding the 2 graphs representing the variation of the malaria case incidence in the Atacora and Alibori-Donga regions being under IRS from 2011 to 2018 as mentioned by the health facilities, there is no reduction (Fig. 4). As a matter of fact, a retrospective investigation was carried out based on the indicators recorded by Atacora and Alibori-Donga health’s centers on the one hand, and malaria incidence calculated by the statisticians of the NMCP using the formula of Kalala et al. [27] and Hellenbrand et al. [28] during the period of IRS on the other hand. The goal of this investigation was to have an idea on the evolution of malaria incidence in the communes where communities were protected by IRS. In addition, a report of a project implemented from 2011 to 2015 in Benin, Cameroon, Kenya, India and Sudan on the impact of vector resistance on the efficacy of vector control tools (LLINs and IRS) showed mitigated results. In this study, the authors found very little correlation between the decrease of entomological parameters and both parasitological and clinical parameters [14, 17]. Another study on the efficacy of four vector control interventions supported by the French Ministry of Cooperation in an area of high vector insecticide resistance in Benin did not show significant differences from one intervention to another [18, 19]. This study concerned a cluster randomized controlled trial carried out in 28 villages southern Benin villages to investigate on the efficacy of various vector control interventions: LLIN targeted coverage to pregnant women and children under 6 years (TLLIN, reference group), LLIN universal coverage of all sleeping units (ULLIN), TLLIN plus full coverage of carbamate-IRS applied every 8 months (TLLIN + IRS), and ULLIN plus full coverage of carbamate-treated plastic sheeting (CTPS) (ULLIN + CTPS). The clinical incidence density of malaria was reduced neither in the children from the ULLIN group nor in those from TLLIN + IRS group nor the ULLIN + CTPS group compared with reference group (TLLIN) despite reduction of HBR and EIR. Relations between *P. falciparum* incidence and EIR had already been observed by Beier et al. [29] in a study in western Kenya showing that measurements of either the EIRs or HBRs can be used to predict corresponding attack rates in children. Some years later, the basic relationship between EIR and *P. falciparum* prevalence accross different ecological zones showed convincingly that substantial reductions in malaria prevalence are likely to be achieved only when EIRs are reduced to levels of less 1 infective bite per person per year [30]. This report showed that some sites with EIRs < 5 infected bites per year had levels of *P. falciparum* prevalence exceeding 40% and when transmission exceeded 15 infected bites per year, there were no sites with prevalence rates < 50%.

To curtail residual malaria transmission, additional interventions able to target vectors escaping IRS should be prioritized. For instance, people who sleep indoors should use LLINs. Those who remain outdoors for various activities (cooking, praying, listening to the radio, eating, doing household chores) should use anti-mosquito ointment or smoke coils. Another alternative would be wearing garments impregnated with repellent insecticides which are available in some countries.

The evolution of malaria incidence in the communes where communities were protected by IRS presented from data collected in the health facilities should be more precise. That is why, the NMCP has decided to implement, from next year, a research study on epidemiological data on *P. falciparum* incidence and prevalence in collaboration with the parasitological research unit of CREC and the Faculty of Health Sciences of the University of Abomey-Calavi, Benin.

#### The choice of the control district affects the estimation of the EIR reduction in the districts under intervention

The high rates of reduction of the entomological parameters mentioned for the districts under IRS should be taken with caution. They were estimated based on the data collected in the control districts. Yet, it is not easy to have control areas with the same environmental characteristics as the intervention areas. The M&E of IRS implemented in Alibori and Donga regions had an advantage. During this monitoring, two periods of the same area were compared: before (2016) IRS for source base and after (2017, 2018) IRS. However, between 2016 and after (2017/2018), other factors might have changed (season, socioeconomic conditions). For the other regions (Ouémé, Atacora), in absence of source data before IRS intervention, not choosing the same areas for both intervention and control was a limit to the study. For these types of control areas, it is difficult to find control sites with characteristics similar to those of intervention sites. The two methods have inconveniences which might have influenced the estimation of EIR reduction rate.

Choosing the best control is an important component. Baseline data could have been collected forone-year before IRS in all regions, but at the beginning of IRS implementation in Benin, particularly in Ouémé region and after in Atacora region, decisions were taken in haste to avoid losing PMI financing. Given that the assessment of IRS impact is in connection with the control zone data, authors of this paper suggest proper arrangements to identify the best control.

### Some key elements of Benin IRS strategic plan

#### Strategy of IRS extension

The National Strategic Plan of Vector Control developed by the National Malaria Control Programme states that IRS should start in some eligible areas and be progressively extended to other areas. What is observed is quite different: instead of a strategy of extension, what occurs is IRS withdrawal and move from one area to another. These moves are all due to the same reason: lack of evident correlation between the decrease of EIR and malaria incidence. To benefit from the epidemiological impact of IRS, it is desirable to redefine the criteria of the eligible areas by prioritizing areas where malaria transmission does not go beyond the period of residual effect of the insecticide.

#### How can IRS in Benin benefit from insecticide resistance management strategies and protocols**?**

The emergence of *An. gambiae* resistance to bendiocarb in Atacora region [4, 11, 13, 18] might have been linked not only to the IRS campaign, but also to the massive use of agricultural insecticides against cotton pests [31]. The management of resistance cannot be perceived by replacing one insecticide with another. Two methods could be used as far as Atacora is concerned:

i. the NMCPs should avoid exposing *Anopheles* to the same product over several years in order to weaken the mosquitoes carrying resistance genes (fitness cost). To achieve this, as proposed by Akogbeto et al. [13], alternation of IRS campaigns with LLIN distribution campaigns should be a way. This means that the implementation of IRS will take place two years after the distribution of LLINs. By that time, most LLINs will have lost their insecticidal effectiveness and some will have been torn. The loss of effectiveness of pyrethroids will then be covered by IRS.
ii. a combination of two insecticides with different modes of action could be used in rotation with the first method. The method based on combination or mosaic use of 2 or 3 insecticides is well known to NMCPs, but its implementation is quite hard due to the limited variety of available products recommended for IRS. However, the release of new IRS insecticides for use (SumiShild of Sumitomo and Fludora Fusion of Bayer) should facilitate the implementation of insecticide resistance management.

### Communication

The withdrawal of IRS for another region is often accompanied by displeasure from the beneficiary communities. This was the case in Ouémé and Atacora. And, this is the reason why, before starting IRS implementation in a region, the beneficiary community should be informed that IRS is not a permanent control measure and might be replaced by another intervention at a specific time. In conclusion, clear communication and expectations are important.

### Financing of IRS intervention

Financing is a key factor. It is an important point for IRS sustainability in Africa. Most of IRS campaigns in this region are supported by PMI and other partners. Unfortunately, IRS in Africa cannot remain at the expense of international partners forever. If for any reasons, the financial partner withdraws, this could rebound in malaria transmission due to IRS termination. This is the case for IRS results in Benin: when IRS was moved from Ouémé to Atacora, and from Atacora to Alibori and Donga, a resurgence of all malaria entomological indicators was noted a year later [3]. As mentioned by Akogbéto et al. [13], IRS must be made cheaper and more affordable. There are irreducible sections included in IRS cost planning. This is the case when environmental compliance, logistics and insecticide provisioning are involved. However, costs related to staff, management and technical assistance due to the presence of international NGOs supporting the implementation must be reduced. African countries should get ready to take over from the financing in areas where IRS gives satisfying results.

### Community practices affecting IRS impact

Dealing with reductions in entomological parameters that have not necessarily been associated with similar reductions in epidemiological indicators in communes under IRS, it is theorized that community behaviours may account for continued high numbers of malaria cases despite the use of both IRS and reported high rates of use of insecticide-treated nets (ITNs). To identify household practices that may impact vector control strategies, a parallel study performed a night time socio-behavioural surveillance during the cold, dry and rainy seasons in three regions where IRS had been done (Alibori, Donga, Atacora) [32]. This investigation showed that during the cold season, 25.1% of household members (HHMs) slept outdoors without net, 23.8% during the rainy season and 41.1% during the hot period. More than 90% of the HHMs in the study were not protected against mosquito bites between 7 PM–10 PM during all the three seasons because most were performing activities outdoors without the protection of IRS, likely due to poor lighting and limited space within their household. During the hot season, the majority of HHMs were not protected at all from mosquito bites after 10 PM because they either slept outside without protection from IRS or slept inside without an ITN. Households were also noted to have large amounts of indoor belongings and clothing hanging on the walls that served as places for mosquitoes to rest other than IRS-treated walls.

### Sleeping under LLINs in the districts under IRS as a supplement malaria control effort

During the 10-year campaigns of IRS, the majority of mosquitoes entering the treated houses were found fed. As a matter of fact, when a mosquito succeeds in entering a treated house, it goes directly to its host and gets its blood meal even when the house is treated. After the meal, the mosquito either remains on the walls or on indoor belongings and clothing hanging on the walls or seeks to escape when the house is treated. This situation was explained since fed mosquitoes were collected by pyrethrum spray catch and in exit window traps in treated houses and experimental huts in Malanville [33], as well as at community level [18, 34].

The question related to the simultaneous use of ITN + IRS was asked several times. There is no doubt that, in Benin, despite IRS, nobody wants to abandon their net. Given the relative high blood feeding rate of mosquitoes in the communes under IRS, authors suggest that the NMCPs educate communities that are protected by IRS, particularly children and pregnant women, to consider sleeping under LLINs to supplement malaria control efforts.

### IRS programme evaluation

M&E is an important component of IRS implementation. The propose of this study is to enlighten the stakeholders involved in vector control, especially PMI countries in Africa, about many challenges related to this intervention. M&E should go along with IRS implementation, but for a limited time. Two 3-year cycles of M&E after a year of source data collection is sufficient for any in-use insecticide.

## Conclusion

In Benin, IRS has demonstrated its effectiveness on the entomological indicators of malaria. From 2008 to 2018, a significant decrease in EIR was noted each year during the period of the residual effect (4 months) of the insecticides used. However, there does not seem to be a correlation between the decline in entomological and epidemiological indicators. Indeed, according to the data recorded in the registers of health facilities, there is no reduction in the incidence of malaria cases in communes under IRS. Several reasons explain the lack of epidemiological evidence impact of IRS:

i. it may be insufficient to decrease epidemiological indicators given that the residual EIR in IRS communes was still higher than it was in some regions of stable malaria. Despite an overall IRS reduction of 90%, the 10% remaining EIR may be above the threshold required to induce a significant decrease in the epidemiological indicators.
ii. since the IRS impact assessment is related to the control zone data, the choice of the control might have influenced the EIR reduction estimation in over-estimating the level, because it is difficult to select control sites with the same environmental characteristics as intervention sites.
iii. half of all mosquitoes that entered IRS-treated houses still succeeded in taking human blood meals.
iv. human behaviour limits IRS efficacy. Recent data show that > 90% of Benin citizens are not fully protected by IRS from 7 to 10 PM; this is due to the fact that they remain outside. Also, most people are not fully protected from mosquito bites after 10 PM because they either sleep outside without IRS protection or inside without an ITN. Besides, people have large amounts of items hanging on walls where mosquitoes rest, avoiding IRS-treated walls.

Despite these arguments, it would be risky to incriminate IRS alone in maintaining the incidence of malaria from 2008 to 2018 in the four regions. During this period, they benefit from other interventions including the management of malaria cases, the use of artemisinin-based combination therapy (ATC), rapid diagnostic tests, and intermittent preventive therapy in pregnant women.

Finally, three other issues are important to consider:

i. Vector resistance management strategies are sometimes poorly understood. Management aims to prevent the emergence of resistance; this is different from replacing an insecticide by another after resistance emerges.
ii. Before IRS begins, communities should be informed that IRS will not be a permanent measure. Clear communication and expectations are important.
iii. African countries should get prepared as to finance IRS and other vector control interventions themselves.

## Data Availability

The data used and/or analysed in this study are available from the corresponding author on reasonable request.

## Abbreviations

IRS: indoor residual spraying
PMI: President’s Malaria Initiative
USAID: United States agency for international development
WHO: World Health Organization
LLIN: long-lasting insecticidal nets
MDGs: millennium development goals
NMCP: national malaria control programme
EIR: entomological inoculation rate
HBR: human biting rate
ELISA: enzyme-linked immunosorbent assay
PCR: polymerase chain reaction
CSP: circumsporozoite protein
EWT: exit traps
HLC: human landing catch
SI: sporozoite index
IRS: indoor residual spraying
PSC: pyrethrum spray catch
CS: micro-encapsulated formulation
CREC: Centre de Recherche Entomologique de Cotonou
EC: emulsifiable concentrate
RTI: research triangle international
ITN: insecticide-treated net
